# Unexpected selections of *Plasmodium falciparum* polymorphisms in previously treatment-naïve areas after monthly presumptive administration of three different anti-malarial drugs in Liberia 1976–78

**DOI:** 10.1186/s12936-017-1747-6

**Published:** 2017-03-13

**Authors:** Irina T. Jovel, Anders Björkman, Cally Roper, Andreas Mårtensson, Johan Ursing

**Affiliations:** 1 Malaria Research, Department of Microbiology, Tumour and Cell Biology, Karolinska Institutet, Stockholm, Sweden; 2 Faculty of Infectious and Tropical Diseases, London School of Hygiene and Tropical Medicine, London, UK; 3 Department of Women’s and Children’s Health, International Maternal and Child Health Unit, Uppsala University, Uppsala, Sweden; 4 Department of Infectious Diseases, Danderyds Hospital, Stockholm, Sweden

**Keywords:** *Plasmodium falciparum*, Malaria, Liberia, Resistance, *pfcrt*, *pfmdr1*, *pfnhe1*, *pfdhfr*, *pfdhps*, *pfmrp1*

## Abstract

**Background:**

To assess the effect on malaria prevalence, village specific monthly administrations of pyrimethamine, chlorproguanil, chloroquine or placebo were given to children in four previously treatment-naïve Liberian villages, 1976–78. *Plasmodium falciparum* in vivo resistance developed to pyrimethamine only. Selection of molecular markers of *P. falciparum* resistance after 2 years of treatment are reported.

**Methods:**

Blood samples were collected from 191 study children in a survey in 1978. Polymorphisms in *pfcrt*, *pfmdr1*, *pfdhfr*, *pfdhps*, *pfmrp1* and *pfnhe1* genes were determined using PCR-based methods.

**Results:**

*Pfcrt* 72–76 CVIET was found in one chloroquine village sample, all remaining samples had *pfcrt* CVMNK. *Pfmdr1* N86 prevalence was 100%. A *pfmdr1* T1069_ACT→ACG_ synonymous polymorphism was found in 30% of chloroquine village samples and 3% of other samples (P = 0.008). Variations in *pfnhe1* block I were found in all except the chloroquine treated village (P < 0.001). Resistance associated *pfdhfr* 108N prevalence was 2% in the pyrimethamine village compared to 45–65% elsewhere, including the placebo village (P = 0.001).

**Conclusions:**

Chloroquine treatment possibly resulted in the development of *pfcrt* 72–76 CVIET. Selection of *pfmdr1* T1069_ACG_ and a *pfnhe1* block 1 genotypes indicates that chloroquine treatment exerted a selective pressure on *P. falciparum.* Pyrimethamine resistance associated *pfdhfr* 108N was present prior to the introduction of any drug. Decreased *pfdhfr* 108N frequency concurrent with development of pyrimethamine resistance suggests a non-*pfdhfr* polymorphisms mediated resistance mechanism.

**Electronic supplementary material:**

The online version of this article (doi:10.1186/s12936-017-1747-6) contains supplementary material, which is available to authorized users.

## Background

*Plasmodium falciparum* has historically shown considerable ability to develop resistance to antimalarial drugs. Increased malaria specific morbidity and mortality have been associated with development and spread of quinine (QN), chloroquine (CQ) and sulfadoxine–pyrimethamine (SP) resistance. Reports of resistance to the currently recommended artemisinin-based combination therapy are consequently of major concern and highlight the importance of understanding the evolution of drug resistance [1–6].

At least fifteen single nucleotide polymorphisms (SNP) in the *P. falciparum* chloroquine resistance transporter (*pfcrt*) gene have been associated with CQ resistance in field isolates [7–9]. Specific haplotypes at positions 72–76 have been linked to regional evolution of CQ resistance and the 76T allele appears essential for resistance [10–12]. Drug resistance associated SNPs have also been identified in the multidrug resistance gene 1 (*pfmdr1*) including N86Y, Y184F, S1034C, N1042D, F1226Y and D1246Y. Various combinations of these SNPs have been shown to modulate levels of drug resistance/tolerance to QN, CQ, amodiaquine, mefloquine and lumefantrine [13].

The *P. falciparum* Na+/H+ exchanger 1 gene (*pfnhe1*) has been associated with QN resistance [14, 15]. Specifically, >1 DNNND repeat and one DDNHNDNHNND repeat in the coding microsatellite ms4760 have been associated with reduced in vivo QN sensitivity [14, 16, 17]. The function of *pfnhe1* has not been fully determined, but it has been proposed that it actively effluxes protons to maintain a pH of 7.4 within the parasite [18]. *Pfnhe1* has also been suggested to play a role in CQ resistance [19, 20], but this was subsequently disputed [21, 22].

Pyrimethamine (PYR) resistance has been associated with polymorphisms A16V, N51I, C59R, S108N/T and I164L in *P. falciparum* dihydrofolate reductase (*pfdhfr*). Typically, an initial S108N change that is considered to be essential for PYR resistance occurs, causing a 7- to 50-fold increase of IC 50 in vitro. Accumulation of additional SNPs further increases IC 50 [23, 24]. Similarly, *P. falciparum* dihydropteroate synthase (*pfdhps*) SNPs S436F/A, A437G, K540E, A581G and A613S have been associated with sulphadoxine resistance. The A437G substitution appears to be the first step for resistance to sulphadoxine and addition of 540E, 436F/A, 581G and 613S further enhance resistance [25]. Quintuple *pfdhfr* 51I/59R/108N and *pfdhps* 437G/540E have been shown to result in highly sulphadoxine–pyrimethamine (SP) resistant parasites [26, 27]. The R1466K SNP of *P. falciparum* multi drug resistance protein 1 (*pfmrp1*) has also been associated with recrudescence after treatment with SP [28].

The aim of this study was to determine possible early *P. falciparum* in vivo selection of genetic markers associated with antimalarial drug resistance after repeated exposure to CQ, PYR or chlorproguanil (CPGN). A large field study conducted between 1976 and 1978 in four villages in northern Liberia where virtually no anti-malarials had been previously used was therefore revisited [29]. Children aged 2–9 years received monthly administrations of CQ, PYR, CPGN or placebo depending upon which village they lived in. One drug was consistently used in each village. During the study period *P. falciparum* developed in vivo resistance to PYR and partially reduced susceptibility to CPGN whilst CQ remained effective [29–31]. The in vivo drug susceptibilities were assessed as Day 7 clearance of parasitaemia by microscopy after single doses of 10 mg/kg CQ, 2 mg/kg PYR or 1.5 mg/kg CPGN respectively. For CQ the clearance rates were 100% among children in CQ village and the control village. For PYR the clearance rates were 7% among children in PYR village and 96% in the control village. For CPGN the clearance rates were 53% among children in CPGN village and 96% in the control village. Sequence variation in *pfcrt*, *pfmdr1*, *pfnhe1*, *pfmrp1*, *pfdhfr* and *pfdhps* in samples collected after 2 years of monthly presumptive treatment with either CQ, PYR, CPGN or placebo were determined.

## Methods

### Study area

The clinical study from which samples were used was conducted in a rural area of northern Liberia 25–35 km from Yekepa. Four villages, about 5 km from each other, were included in the study: Bondi, Baytonwee, Bonah and Kinon. Each village had around 500 inhabitants. The villages were separated by dense forest, which made access to and between the villages difficult. Access to healthcare and specifically anti-malarial treatment was very limited when the study started in 1976. The climate is tropical with a dry season from November to April and a rainy season from May to October. The temperature is generally between 21 and 32 °C. Malaria was holoendemic when the clinical study was conducted [29, 30]. In 1976 (prior to deployment of monthly presumptive treatment) the overall parasite prevalence in the study area assessed by blood slide microscopy in 2–9 years old children was 82% for *P. falciparum*, 39% for *Plasmodium malariae* and 9% for *Plasmodium ovale* [32].

### Study group, consent, drug administration, sample collection and storage

Children 2–9 years old, present in the villages in November 1976 were invited to participate in the study. All (n = 282) agreed to participate after verbal informed consent. The four villages were visited every 14 days for 3 years. Children were given single dose anti-malarial drugs every 4 weeks for 2 years as follows. Bondi: 8–15 mg base/kg dose of CQ. Baytonwee: 1.0–2.0 mg base/kg of CPGN. Bonah: 1.3–2.5 mg base/kg of PYR. Kinon: 1 or 2 tablets of Vitamin B (placebo). Drug intake was supervised by the investigators [29].

Children in all villages were also treated with 8–15 mg/kg base of single dose CQ in case of fever and confirmed malaria infection throughout the two-year study period. At the time this was considered a therapeutic dose of CQ [29]. After 2 years of monthly presumptive treatment, capillary whole blood samples were collected 4–6 weeks after last intake of study drug (i.e. from 28th of February to 17th of March, 1978) from all children (n = 191) present and still included in the study. The children were now 4–11 years old. Samples were coded and stored at −20 °C. The prevalence of *P. falciparum* in each treated village, as assessed by microscopy, was 23% (CQ), 94% (PYR), 73% (CPGN) and 88% (placebo) [29], whereas *P. malariae* and *P. ovale* were rarely detected.

The study was approved by the Liberian Institute of Biomedical Research [29]. Molecular analyses were approved by the Stockholm regional ethics board (reference number: 2013/836-32).

### Molecular analysis

DNA was extracted using QIAamp Blood Mini Kit (QIAgen Biosciences, Germantown, MD, USA). The samples were extracted separately from other samples in our research laboratory to minimise the risk of contamination. Extracted DNA was stored at −20 °C until use.

*Pfcrt* 72–76, *pfmdr1* 1034–1246, *pfdhfr* 16–185, *pfdhps* 436–632 and *pfnhe1* ms4760 haplotypes were identified by PCR amplification followed by sequencing using previously described PCR protocols [12, 16, 33–35]. The Sequencher™ software version 4.6 (Gene Codes Corporation, Ann Arbor, MI, USA) was used for sequence analyses. The *P. falciparum* 3D7 clone sequence obtained from the NCBI database was used as references for *pfcrt* (Accession no. NC_004328), *pfmdr1* (Accession no. XM_001351751.1), *pfdhfr* (Accession no. XM_001351443.1) *pfdhps* (Accession no. XM_001349382.1) and *pfnhe1* (Accession no. XM_001349726). *Pfnhe1* ms4760 sequences were also compared with previously described isolates and clones [16, 36].

In addition to sequencing PCR–RFLP (restriction fragment length polymorphism) was used to identify the following SNPs; *pfcrt* K76T, A220S, Q271E, N326D/S, I356L/T, R371I, *pfdhfr* N51I, C59R, S108T/N, *pfdhps* S436F/A, A437G and K540E and *pfmdr1* N86Y and Y184F using previously described methods [12, 37, 38]. The *pfmrp1* allele K1466R was determined by pyrosequencing [28].

PCR and restriction products were resolved on 2% agarose gels (Amresco, Solon, OH, USA). All gels were stained with a nucleic acid gel stain (GelRed™, Biotium Inc. Hayward, CA, USA) and visualized under UV transillumination (GelDoc®, Biorad, Hercules, CA, USA). PCR products were purified and sequenced commercially (Macrogen Inc. Seoul, Korea).

### Statistics

Data were entered, validated and analysed on Microsoft Excel 2003 and StataCorp 12. Allele proportions were calculated by dividing the number of samples with a certain allele by the number of samples with an identifiable allele at that position. Thus mixed infections contributed to the proportion of both alleles. Associations were determined using Fishers Exact test using StataCorp 12.

## Results

A total of 191 samples were included in the study, of which 50, 48, 48 and 45 were from the CQ, PYR, CPGN and placebo village, respectively. At least one SNP was identified in all samples (not only microscopy positive) and each SNP was identified in 81–98% of samples. Negative controls were used throughout without any signs of contamination.

### Pfcrt

*Pfcrt* K76 was found in 185/186 (99%) samples. A single sample carrying *pfcrt* 76T was detected in the village using CQ. The CVMNK haplotype was found in 177/178 (99%) successfully sequenced samples and the CVIET haplotype was found in the sample that had *pfcrt* 76T by RFLP. The *pfcrt* CVIET result was confirmed by PCR–RFLP and sequencing after re-extracting the sample alone. The *pfcrt* haplotype at codons 220, 271, 326, 356 and 371 was determined in the CQ and placebo villages only. The wild type AQNIR haplotype was found in all successfully amplified samples (88/95 [93%]) including the sample with the *pfcrt* CVIET haplotype.

### Pfmdr1

*Pfmdr1* N86 was found in all 161 (100%) successfully amplified samples. *Pfmdr1* Y184 was found in 75/160 (47%), 184F in 47/160 (29%) and Y184+184F in 38/160 (24%). The ratio of 184F was non-significantly higher in the CPGN (19/37 [51%]), PYR (27/46 [59%]) and CQ (19/33 [58%]) villages compared to the placebo village (20/44 [45%]). Similarly, no significant difference was observed in Y184 ratio in the CPGN (22/37 [59%]), PYR (32/46 [70%]), CQ (20/33 [61%]) villages compared with the placebo village (29/44 [66%]). Excluding mixed infections did not alter the findings significantly.

The *pfmdr1* S1034C, N1042D, F1226Y and D1246Y haplotypes were SNFD in all 163 (100%) successfully sequenced samples. The frequency of a synonymous SNP at codon 1069 (ACT→ACG) was found to be significantly higher in the CQ village (11/17 [30%]) compared to the PYR (3/45, P = 0.008), CPGN (1/38, P = 0.001) and placebo (1/43, P = 0.001) villages. Three novel synonymous SNPs 1061 (GAT→GAC), 1137 (TCA→TCC) and 1185 (ATT→ATC) were found in 1, 4 and 2 of the 163 samples, respectively. Two novel non synonymous SNPs 1166 (GGA→GCA) and 1190 (GAT→AAT) were found in 2 and 1 samples, respectively.

### Pfnhe-1

*Pfnhe1* ms4760 was successfully amplified in 156/191 (82%) samples. Eight of the 114 previously described *pfnhe1* haplotypes [16, 36] and 47 novel ms4760 haplotypes were found. The haplotypes found are shown in Additional file 1: Table S1. Of the 47 new haplotypes, 32 consisted of variation in Block I. Proportions of samples with haplotypes in block I are presented in Table 1. Variation in *pfnhe1* block I were significantly more common in the villages using CPGN (9/35 [26%], P < 0.001), PYR (18/44 [41%], P < 0.001) and placebo (16/39 [41%], P < 0.001) compared to the CQ treated village (0/38). Though Block I did not vary in the CQ village 10 different ms4760 haplotypes were identified due to variations in other parts of ms4760.

**Table 1:** *Pfnhe*-*1* ms4760 types

ms4760 haplotype	Chlorproguanil	Pyrimethamine	Chloroquine	Vitamin B	Total
Number of previously described ms4760 haplotypes with no variation in block I^a^	22	25	25	17	89
Number of novel ms4760 haplotypes with no variation in block I^b^	4	2	*13* ^*d*^	6	25
Number of novel ms4760 haplotypes with variation in block I^c^	9	17	*0* ^*e*^	16	42
Total no. of different haplotypes	13	29	10	23	55
Total no. of samples	35	44	38	39	156

Resistance associated alleles are shown in italics

^a^Previously described ms4760-1, -3, -4, -5, -7, -28, -98 and Mali_15 with no variation in block I

^b^ms4760 haplotypes Lib_01 to Lib_15 with no variation in block I

^c^ms4760 haplotypes Lib_19 to Lib_47 with variation in block I

^d^The frequency of samples with no variation in block I was higher in the CQ treated village compared with the frequencies in the villages treated with CPGN (P = 0.028), PYR (P = 0.001), placebo (P = 0.068) and with pooled data from all villages (P = 0.001)

^e^The frequency of samples with variation in block I was lower in the CQ treated village when compared with separate and pooled frequencies from the other villages (P < 0.001)

The ratio of ms4760 haplotypes in successfully amplified samples were significantly lower in the villages using CQ (10/38 [26%], P = 0.006) but non-significantly lower in the villages using CPGN (13/35 [37%], P = 0.07) and PYR 19/44 (43%, P = 0.19) compared to placebo 23/39 (59%).

The number of quinine resistance associated DDNHNDNHNND repeats in block V were one in 30/156 (19%), two in 124/156 (80%) and 3 in 2/156 (1%) samples. The distributions of repeats were similar in all villages. There were no associations of variations in block I or V and any SNPs in *pfmdr1, pfdhfr or pfdhps*.

### Pfdhfr

Allele proportions are presented in Table 2. The proportion of the PYR resistance associated 108N was significantly lower in the village using PYR (1/46 [2%]) compared to the villages using CPGN (17/39 [44%], P < 0.001), CQ (18/39 [46%], P < 0.001) and placebo (29/45 [64%], P < 0.001). The other codons found were all A16, N51, C59 and I164 that are associated with sensitivity to PYR.

**Table 2:** Allele frequencies at resistance associated *pfdhfr* codons 16, 51, 59, 108 and 164

Village	A16	N51	C59	S108	108*N*	108*S*+*N*	I164
Chlorproguanil	36/36	36/36	36/36	21/39 (54%)	17/39 (45%)	1/39 (3%)	39/39
Pyrimethamine	41/41	41/41	41/41	45/46 (98%)	1/46 (2%)^a^	0	44/44
Chloroquine	31/31	31/31	31/31	21/39 (54%)	18/39 (46%)	0	36/36
Vitamin B	39/39	39/39	39/39	15/45 (33%)	29/45 (65%)	1/45 (2%)	40/40

Resistance associated alleles are shown in italics

^a^The frequency of samples with the resistance associated *pfdhfr* 108N was lower in the pyrimethamine treated village when compared with separate and pooled frequencies from the other villages (P < 0.001)

### Pfdhps

Allele proportions are presented in Table 3. The sulphadoxine resistance associated *pfdhps* 613S was found in 10% (14/135) of samples in this sulphadoxine–pyrimethamine naïve area. No other resistance associated polymorphisms were found. The proportion of *pfdhps* 613S was significantly lower in the PYR village (0/38) compared to the village using CQ (7/26 [27%], P = 0.001) and non-significantly lower in the villages using CPGN (3/31 [10%], P = 0.09) and placebo (4/39 [10%], P = 0.12). When data from the CPGN, CQ and placebo villages were pooled the findings were significantly different compared with the PYR village (P = 0.01).

**Table 3:** Allele frequencies at resistance associated *pfdhps* codons 436, 437, 514, 540, 581 and 613

Village	S436	A437	I514	K540	A581	A613	*613* *S*
Chlorproguanil	40/40	40/40	32/32	32/32	32/32	29/32 (91%)	3 (9%)
Pyrimethamine	38/38	38/38	38/38	38/38	38/38	38/38	*0* ^a^
Chloroquine	30/30	30/30	26/26	26/26	26/26	19/26 (73%)	7(27%)
Vitamin B	40/40	40/40	40/40	40/40	40/40	35/39 (90%)	4 (10%)

Resistance associated alleles are shown in italics

^a^The frequency of samples with the resistance associated *pfdhps* 613S was lower in the pyrimethamine treated village compared to pooled frequencies from the other villages (P < 0.01) and the village using CQ (P = 0.001)

### Pfmrp1

*Pfmrp1* R1466 (wild type) was found in 137/137 (100%) successfully amplified samples.

## Discussion

This study provides a unique insight into the early appearance of genetic markers associated with anti-malarial drug resistance and their selection 2 years after monthly presumptive treatment with CQ, PYR and CPG or placebo in a previously virtually anti-malarial treatment-naïve area of Liberia.

One sample had the *pfcrt* 76T SNP in the 72-76 CVIET haplotype not previously recorded in Africa as early as 1978. *Pfcrt* CVIET was confirmed by repeated PCR analyses on re-extracted DNA several months after the first analyses. Contamination is, therefore, unlikely to explain this remarkable finding. Throughout the world *pfcrt* 76T has been linked to CQ resistance within various *pfcrt* 72–76 haplotypes. By 1978, *pfcrt* CVIET had existed in Southeast Asia for many years but not in Africa. Molecular analysis has shown that the CVIET later found throughout Africa was derived from this Southeast Asian lineage [8]. Southeast Asian *pfcrt* typically have additional downstream SNPs at codons 220, 271, 326, 356 and 371. Our sample had those typical among African origin CQ sensitive genotypes (and 3D7) [39]. Furthermore, the study area was very isolated making importation unlikely. The most likely explanation is thus that *pfcrt* 72–76 CVIET arose in the CQ study village de-novo following a period of intermittent presumptive treatment with CQ.

Irrespective of how this isolated CVIET haplotype came to be in the study area it did not seem to have become widespread as only *pfcrt* CVMNK parasites were found in 50 samples collected 3 years later in the same area [40]. This observation is consistent with longitudinal surveillance of high in vivo efficacy and in vitro susceptibility to CQ in Bondi (CQ village) between 1976 and 1978 [29, 41]. In the ordered evolution of *pfcrt* SNPs that eventually give rise to CQ resistance described by Summers et al. [42], having CVIET alone with no other *pfcrt* SNPs only resulted in a small increase in Pfcrt’s ability to transport CQ and hence to confer resistance [42]. Furthermore, these mutations interfere with haemoglobin digestion resulting in a loss of fitness [43]. Thus CVIET might have developed in Liberia but did not become widespread as it provided only weak selective advantage in this holoendemic area.

The increased frequency of the synonymous SNP *pfmdr1* T1069_ACG_ and decreased diversity in *pfnhe1* block I in the CQ treated village indicates that CQ exerted a selective pressure on the drug-naïve parasite population. In line with this both genes have previously been linked to CQ resistance [19, 20] and previously described block I ms4760 haplotypes in samples collected after the spread of CQ resistance had the same block I type as that selected for in the CQ village [36]. However, as *pfmdr1* T1069_ACG_ is a synonymous SNP it was not selected itself but is probably a marker for selection of a parasite strain or a *pfmdr1* genotype that had a survival advantage when CQ was used.

Decreased in vitro and in vivo QN susceptibility has been associated with the *pfnhe1* ms4760-1 haplotype that has two DNNND and one DDNHNDNHNND and with having more than one DNNND repeat and one DDNHNDNHNND repeat in some studies but not in others [14, 16, 36, 44]. Though QN was not used in the Yekepa area at the time of this study the frequency of *pfnhe1* ms4760-1 (26%) was similar to the frequency found at day 0 in a recent study in Mali where ms4760-1 was associated with decreased QN susceptibility [16]. Considering the geographic separation of Liberia and Mali results should be compared with considerable caution. However, similar frequencies found in Liberia in 1978, prior to a drug selective pressure and in Mali and Senegal several decades later suggest that ms4760-1 has not been under a strong selective pressure.

The PYR resistance associated *pfdhfr* 108N was surprisingly not selected for in the PYR village despite development of in vivo resistance to PYR within a year of initiating monthly intermittent presumptive therapy [29]. In contrast it was found at significant frequencies in the placebo and CQ villages where no antifolate drugs had been used, suggesting that it was a wild type in Liberia in 1978. Similarly, resistance associated *pfdhfr* 51I, 59R, 108N were found in 9/66 samples collected in The Gambia in 1984 prior to widespread use of SP [45]. These intriguing results suggest that there may have been an alternate mechanism of PYR resistance in our study village as has previously been suggested [13, 46]. Furthermore, the 108N did not provide a selective advantage in the presence of this alternate mechanism but rather the opposite. Amplifications of the *pfdhfr* or GTP-cyclohydrolase (*gch1*) genes have been linked to PYR resistance in the past [47, 48]. Gene amplifications could have occurred in Liberia and might thus constitute an early method of PYR resistance in line with the correlation between *pfmdr1* amplifications and drug resistance to mefloquine and artemisinin derivatives [49]. An alternative mechanism of PYR resistance might be a greater influx of folate into the parasite as has been suggested to be mediated by *pfmrp1* 1466K [28]. However, in Liberia only *pfmrp1* R1466 was found. Concerning *pfdhps*, PYR is not likely to directly exert a selective effect, yet the frequency of *pfdhps* 613S (that is related to sulphadoxine resistance) was lower in the PYR treated village. Perhaps this represents an indirect effect of selection of other genes.

Chlorproguanil (CPGN) is believed to be a DHFR inhibitor that is partly metabolized to the probably more *P. falciparum* active chlorcycloguanil in vivo. In line with this parasites with *pfdhfr* 16V and 108T and 108N have been associated with decreased susceptibility to the structurally similar cycloguanil in vitro and ex vivo, [26, 50–52]. Finding no difference in *pfdhfr* 108N frequency in the CPGN village compared with the placebo village indicates that 108 N was not the cause of the partly reduced CPGN susceptibility seen in the study area [29].

## Conclusion

Unique data on the effect of monthly intermittent presumptive CQ, PYR and CPGN therapy on selection of known resistance genes in previously treatment-naïve *P. falciparum* populations is presented. The *pfcrt* 72–76 haplotype CVIET was detected in the CQ village and possibly developed de-novo. However, none of the downstream *pfcrt* SNPs commonly found throughout CQR genotypes in Africa today were detected. Decreased variation of *pfnhe1* was seen and a synonymous *pfmdr1* 1069T SNP was selected detected in the CQ village suggesting that CQ exerted a selective pressure on the drug naïve parasite population. The PYR resistance associated SNP *pfdhfr* 108N was highly prevalent in this antifolate naïve parasite population and intriguingly deselected concomitant with emergence of PYR resistance suggesting alternative mechanisms of PYR resistance. The results provide new insights into the evolution of anti-malarial drug resistance in Africa.

## Additional file

**Additional file 1: Table S1.** Alignment of pfnhe1 ms4760variants found in Liberia in 1978 (Jovel et al. [40]).
